# Development of Self-Powered Energy-Harvesting Electronic Module and Signal-Processing Framework for Wearable Healthcare Applications

**DOI:** 10.3390/bioengineering11121252

**Published:** 2024-12-11

**Authors:** Jegan Rajendran, Nimi Wilson Sukumari, P. Subha Hency Jose, Manikandan Rajendran, Manob Jyoti Saikia

**Affiliations:** 1 Biomedical Sensors & Systems Lab, University of Memphis, Memphis, TN 38152, USA; 19.jegan@gmail.com; 2 Biomedical Engineering Department, Karunya Institute of Technology and Sciences, Coimbatore 641114, Tamil Nadu, India; nimiwsrec@gmail.com (N.W.S.);; 3 Electrical Engineering Department, Einstein College of Engineering, Tirunelveli 627012, Ramil Nadu, India; 4 Electrical and Computer Engineering Department, University of Memphis, Memphis, TN 38152, USA

**Keywords:** biosensor, ECG signal processing, feature extraction, energy harvesting, machine learning algorithms, physiological vital parameters, wavelet packet analysis

## Abstract

A battery-operated biomedical wearable device gradually assists in clinical tasks to monitor patients’ health states regarding early diagnosis and detection. This paper presents the development of a self-powered portable electronic module by integrating an onboard energy-harvesting facility for electrocardiogram (ECG) signal processing and personalized health monitoring. The developed electronic module provides a customizable approach to power the device using a lithium-ion battery, a series of silicon photodiode arrays, and a solar panel. The new architecture and techniques offered by the developed method include an analog front-end unit, a signal processing unit, and a battery management unit for the acquiring and processing of real-time ECG signals. The dynamic multi-level wavelet packet decomposition framework has been used and applied to an ECG signal to extract the desired features by removing overlapped and repeated samples from an ECG signal. Further, a random forest with deep decision tree (RFDDT) architecture has been designed for offline ECG signal classification, and experimental results provide the highest accuracy of 99.72%. One assesses the custom-developed sensor by comparing its data with those of conventional biosensors. The onboard energy-harvesting and battery management circuits are designed with a BQ25505 microprocessor with the support of silicon photodiodes and solar cells which detect the ambient light variations and provide a maximum of 4.2 V supply to enable the continuous operation of an entire module. The measurements conducted on each unit of the proposed method demonstrate that the proposed signal-processing method significantly reduces the overlapping samples from the raw ECG data and the timing requirement criteria for personalized and wearable health monitoring. Also, it improves temporal requirements for ECG data processing while achieving excellent classification performance at a low computing cost.

## 1. Introduction

Noninvasive devices with sensor interfaces are becoming increasingly popular for monitoring patient health, including heart rate, blood pressure, oxygen saturation, and other important indicators. The application of wearable technology and biosensing in the healthcare network has drawn the attention of numerous researchers [1]. As a result, wearable smart devices with sophisticated signal-processing capabilities will be stepping out, enabling the analysis and monitoring of physiological data in the process of diagnosing various diseases. Over the past ten years, there has been a significant increase in the application of biological signals in a variety of scientific fields, including the military, robotics, telemedicine, cloud computing, activity recognition, and remote monitoring [2,3,4]. The market need for tracking a person’s health state at home is predicted to drive significant growth in the use of wearable bio-health monitoring devices which are being used slowly so far. Numerous studies have been conducted in close relation to the creation of ECG measurement devices and the problem statement has been examined in relation to the combination of wearable technology, digital circuits, sensor integration, signal processing, energy harvesting, and the measurement and monitoring of vital parameters [3,4,5,6,7,8,9,10,11].

Wearable electronic devices have been developed during the last decade to collect bio signals from the body’s surface for use in a variety of biomedical applications. Based on the findings, it was determined that optimal acquisition signal quality is required for the precise measurement of vital parameters and the identification of cardiovascular disorders [11]. The acquisition of a real-time ECG signal for continuous monitoring is still a challenging task and needs to be improved. The long-term monitoring of patients’ chronic conditions at home is a significant barrier for healthcare practitioners because of device power consumption and onboard battery strength. In order to predict and identify a growing number of cardiovascular diseases, there has been an increase in interest recently in developing smart biosensors, which will offer an efficient means of monitoring vital patient parameters like blood pressure, respiration rate, heart rate variability, and heart rate [12]. Heart rate variability (HRV) is a highly significant vital parameter in patients because it raises the risk of serious health problems, including all heart-related issues. According to the research, the number of people with cardiovascular disease is rising and in 2016, this condition claimed the lives of around 17.9 million people. Therefore, the first screening for health concerns at home is crucial and should be considered the best strategy for personalized health monitoring. For the purpose of detecting these diseases early, it is imperative that each person’s heart signal be monitored at home using smart devices with biosensors.

Numerous low-cost medical gadgets have been developed recently to monitor a wide range of important indicators [13]. Additionally, there is proof that using these wearable devices at home, which allow for prompt patient health monitoring, lowers the death rate [14]. Unfortunately, it does not support the long-term continuous monitoring and detection of ECG problems. The development of a device that can monitor aberrant health conditions in the course of daily activities and offers continuous monitoring capabilities is crucial [15]. The most important information can be obtained from an ECG signal’s brief duration, although it might be difficult to identify cardiac arrhythmias in such a short amount of time [16]. The presence of cardiac arrhythmia in the ECG signal during the early phases must be assessed well in advance. According to the research, it is difficult to identify cardiac arrhythmias from an acquired ECG signal in the early stages of heart illness and requires extra care in order to identify and categorize the arrhythmias. There are various techniques for detecting cardiac arrhythmias. In order to identify arrhythmias, the first technique makes use of ECG features that are taken from an ECG signal and fed into hybrid classifiers [17]. But the precision of the classification using this approach becomes crucial, and it mostly depends on the quality of the features that are extracted. Therefore, for a reliable assessment of cardiac arrhythmia, an ECG measurement system with sophisticated signal-processing and adaptable categorization techniques is required. The alternative approach uses the raw signals as the classifiers’ input directly in order to detect arrhythmias. In this case, the classifiers are taught to automatically pick up the features. One of the main limitations of these methods is the high computing complexity required to acquire a quality ECG signal due to the enormous number of processes, which further limits real-time detection performance [18]. One type of potentially fatal illness that has a major impact on people’s health is cardiovascular disease. Using sophisticated signal-processing algorithms to quickly detect arrhythmias can be a useful strategy for the early prevention of cardiovascular illnesses. The goal of the proposed study is to design an autonomous electrocardiogram (ECG) device with an integration of an onboard battery management unit for continuous operation, a multi-level wavelet packet decomposition framework for measuring vital parameters, and a random forest architecture with deep decision trees for signal classification. This paper’s primary contributions are as follows:

1. Finding an alternate solution which uses a flexible approach to power the portable biomedical device for continuous personalized health monitoring applications.
2. The developed prototype facilitates the harvesting and saving of energy from an ambient light environment by using a series of silicon photodiodes and solar cells.
3. The proposed device also gives the flexibility to charge the secondary battery for long-term monitoring purposes during an emergency.
4. The integration of an ECG sensing module, a pre-processing module, wavelet techniques, and hybrid classifiers.
5. Accurate measurement and signal classification which reduces the computational complexity of ECG signal processing, improving classifiers accuracy.

This paper comprises three sections: Section 2 of this paper gives the background of the existing and related works on ECG signal processing and classifications; Section 3 provides a proposed hardware design and methods for a self-powered energy-harvesting module; Section 4 presents the algorithms for ECG signal feature extractions and vital parameters measurement and ECG classification; Section 5 provides experimental results and their discussion; Section 6 concludes the paper.

## 2. Materials and Methods

The increasing requirement for ECG data processing, automated cardiac arrhythmia identification, and continuous monitoring has prompted several researchers to look into this recently. This section of the paper presents the functional materials and methods adopted for ECG signal processing and feature extraction through the state of the art developed device.

### 2.1. ECG Signal Processing

**Figure 1:**
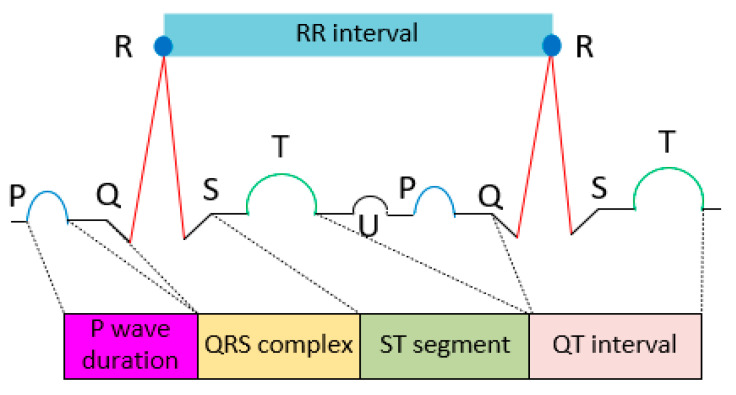
The representation of electrocardiogram signal.

The ECG signal depicted in Figure 1 is recorded to evaluate quick changes in distinctive patterns such as P wave amplitude, QRS complex amplitude, RR interval, ST segment, and QT interval. The ECG signal provides the electrical waves that occurred during the heartbeat. The ECG waveform exhibits peaks and valleys. The depolarization of the atrium and ventricles are represented by P waves and QRS segments, the interval from the start of the QRS complex until the T wave ends is expressed by the QT interval, and the interval from an end of the QRS complex and the start of the T wave is expressed by ST segments. The ECG signal can be processed using a variety of signal-processing techniques, including filtering [19], detrending [20], and wavelet analysis [21] to extract these distinctive patterns. These signal-processing techniques provide the advantages of quick processing, enhanced signal quality, and precise detection. The biomedical signal-processing technique is crucial in obtaining the required information at a specific interval for accurate calculations, and it offers a great deal of flexibility. According to earlier studies, a variety of algorithms including machine learning and sophisticated signal-processing techniques can successfully diagnose many diseases early on, enabling patients to obtain life support on schedule. However, effort must be given to create algorithms that are light, reduce processing times, have low computing costs, and increase classification accuracy.

### 2.2. ECG Noise Removal Methods

Noise interference is one of the main challenges in identifying the different elements from an ECG signal. As seen in Table 1, three primary noises associated with the same frequency range of an ECG signal are baseline wandering, powerline interference, and motion artifacts. An ECG signal’s frequency range is 0.05 Hz to 150 Hz. Motion artifacts can be caused by patient movement [22], baseline wandering, a low frequency noise signal resulting from respiration and electrode impedance changes [23], and powerline interference brought on by the stray effect of alternating current fields due to loops created in patient-used cables [24].

The original signals are being significantly degraded by these noises. Consequently, it is advised that these noises be removed prior to processing and making a diagnosis. Researchers frequently employ several techniques, including adaptive filtering [25], wavelet transform [26], and digital filtering [27] to eliminate unwanted noises. Additional benefits of adaptive filtering include low computational complexity, ease of hardware implementation, and simplicity of use. Researchers could employ digital filtering techniques such as a low-pass filter or a band-pass filter to remove low- and high-frequency noises. The wavelet-based techniques removed the noise by using various mother wavelets [28]. Low complexity and the ability to extract time and frequency information from the input signals are two benefits of wavelet-based techniques. Based on current findings, it has been shown that, in order to effectively remove noise, the mother wavelet used for the wavelet transform strategy should have a strong correlation and the same shape as the input signal.

### 2.3. ECG Features

The automatic detection and classification of cardiac arrhythmias is another important function of the ECG’s characteristics. In essence, the time and frequency domains are used to classify the ECG’s features. Furthermore, this study indicated that certain traits can be obtained from temporal or frequency attributes. Atrial and ventricular responses to cardiac activity can be used to study these characteristics. The important characteristics that could be gleaned from the human heart’s activity are compiled in Table 2.

It was noted from the literature review that the RR interval is one of the most crucial characteristics of the ECG [29]. The R peak can also be used as a reference point to locate other wave patterns. Thus, for the purpose of determining the distinguishing features of numerous diseases, it is imperative to determine the exact location of R peaks and RR intervals. Additionally, the most important factor in the diagnosis of disease is QRS detection [30]. The features from the RR interval that are used for detection and classification are shown in Table 3 below.

### 2.4. Machine Learning for Classification

The two main categories of machine learning approaches are supervised and unsupervised learning. Many methods for supervised and unsupervised learning have been developed for the purpose of predicting and classifying anomalies in ECG data. But for accurate classification, choosing the right classification method becomes crucial and requires more thought. In the field of health sciences, statistical machine learning techniques are crucial for the categorization and detection of numerous illnesses. The process of classifying diseases involves appropriate training with inputs that are well-defined datasets [31]. The process of choosing a machine learning algorithm is mostly based on the availability of input datasets and other signal characteristics. This paper presents a variety of machine learning algorithms to find the best classifier for the identification of aberrant ECG readings. Many approaches for classifying and detecting ECG abnormalities have been studied by numerous researchers and the results are compiled in Table 4.

## 3. Design Criteria for Self-Powered Energy-Harvesting Module

In this section, the proposed development of a self-powered ECG device with the integration of an energy-harvesting module is presented. The proposed module consists of two blocks, which includes an analog front-end unit for ECG signal acquisition and an energy-harvesting module for enabling self-powering capabilities.

### 3.1. Design of Analog Front-End Unit for ECG Device

**Figure 2:**
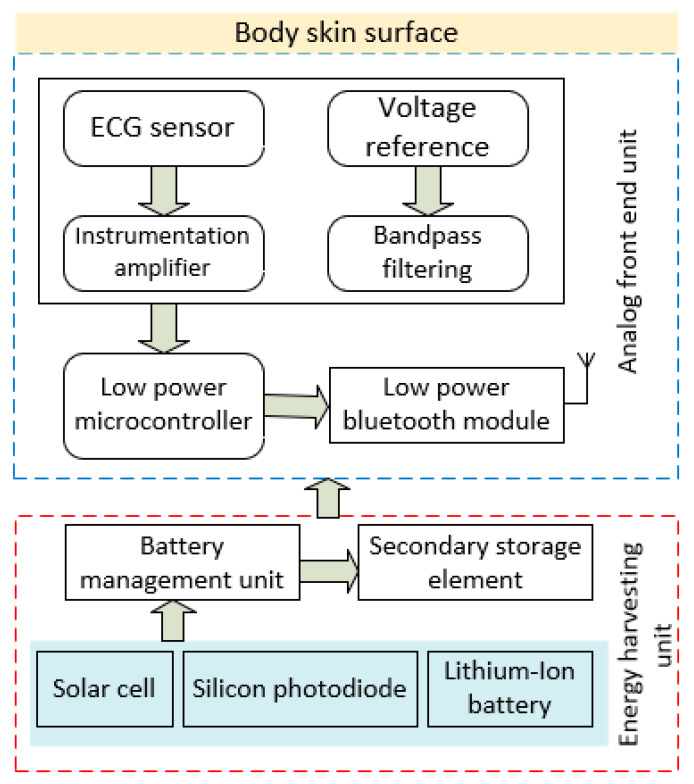
The functional blocks of proposed electronic module hardware elements.

The construction of a fully customizable, self-powered electronic module is described in this section of the paper. The block diagram of the completely integrated, self-powered electronic module is displayed in Figure 2. The developed energy-harvesting module provides flexible approaches for delivering power to the entire device. The energy has been extracted from an ambient light by an array of silicon photodiodes which deliver the power to the device. This device offers a variable powering solution and makes it simple to obtain real-time ECG signals. Additionally, the developed device offers a wireless communication protocol interface for monitoring. A sensor for obtaining an ECG signal is included in the analog front-end electronics unit. A bio amplifier with adjustable gain is used to amplify the low-range signals, and a two-stage filtering unit is used to remove noises. The proposed device consists of different hardware modules in which an analog front-end electronic unit contains a sensor for acquiring an ECG signal, a bio amplifier with adjustable gain for amplifying the low amplitude signal, a two-stage filtering unit for removing the noises, a battery management unit for energy harvesting [45,46], a low power microcontroller and Bluetooth modules for transmitting the data, and a data logging unit for storing the history of data for further offline processing.

#### ECG Signal Acquisition and Characteristics

The traditional ECG instrument captures the heart’s electrical activity [47,48]. By positioning the electrode at a certain spot on the body above the skin, these electrical activities can be detected. In this work, the real-time ECG signal is obtained through the use of ECG electrodes. The various wave patterns from P, Q, R, S, and T waves provide important information for the diagnosis of heart problems [49]. The origins and distinguishing characteristics of the ECG signal are listed in Table 5.

### 3.2. Design of Bio Amplifier

**Figure 3:**
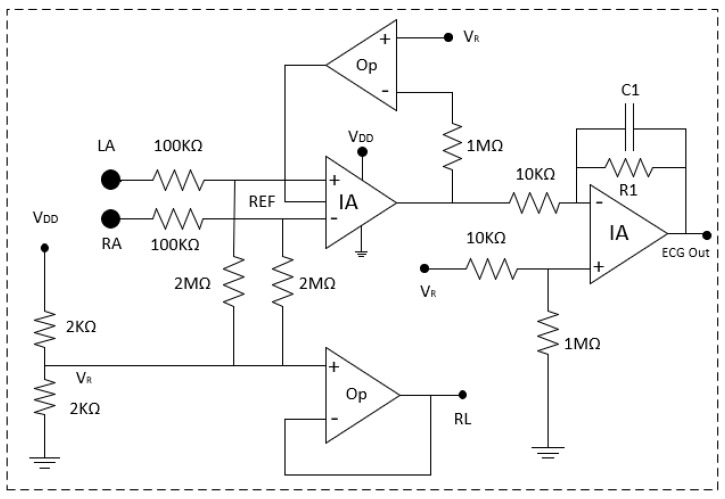
The circuit configuration of ECG bio amplifier.

The low-range ECG signal is intended to be amplified using a low-power CMOS instrumentation amplifier circuit. The entire specification of the instrumentation amplifier spans the supply voltage range of +2.7 V to +5.5 V. By connecting INA 332’s gain pin to its sixth pin, the instrumentation amplifier’s internal gain can be adjusted to five. Two resistors, Ra and Rb can be added between the instrumentation amplifier’s fifth, sixth, and first pins to change the gain of the device. The instrumentation amplifier gain is determined by the Ra to Rb ratio. Figure 3 depicts the suggested ECG amplifier’s circuit layout.

### 3.3. Design of Battery Management Unit

**Figure 4:**
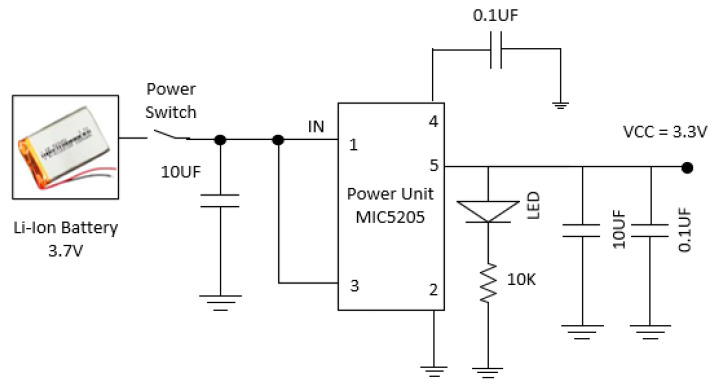
Schematic of power supply unit.

The device is powered by a 3.7 V lithium-ion battery. The power unit’s design blocks are displayed in Figure 4. The created device’s whole module is powered by 3.3 V. As a result, the circuit of the power unit is made to convert 3.7 V to 3.3 V. In this case, the voltage is regulated to 3.3 V using a surface mount chip called the MIC5205 low-dropout voltage regulator. The lithium-ion battery supplies 3.7 V to the first pin of the MIC5205 chip, which has five pins. The created device’s ON/OFF status is controlled by the power switch, which is interfaced between the MIC5205 and the lithium-ion battery. Figure 4 shows the power supply unit design blocks using lithium-ion batteries.

**Figure 5:**
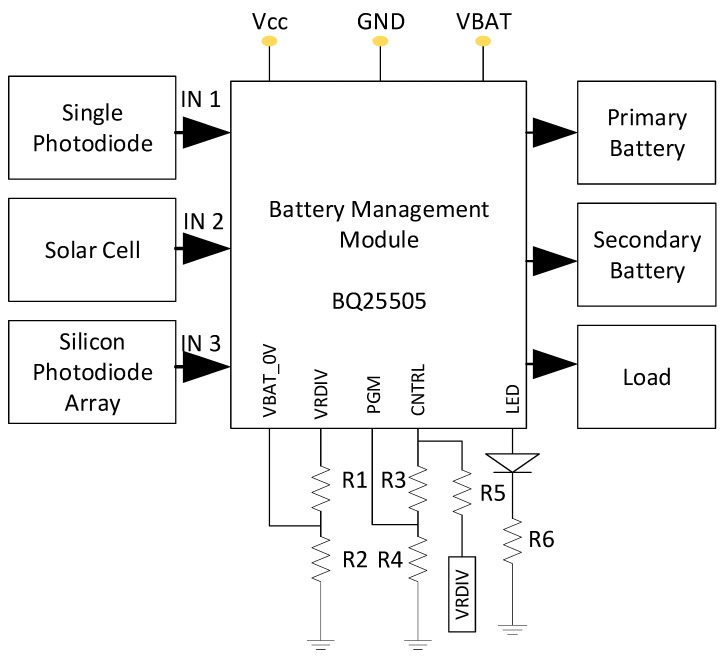
Design blocks of battery management unit.

Figure 5 depicts the block diagram of the battery management unit. The ultra-low-power boost charger with the battery management IC BQ25505 is the core component of the battery management unit design. The circuit architecture allows to produce a steady output voltage regardless of the input voltage. Limiting the output overload voltage is one of the designed battery management unit’s additional benefits. The battery management unit’s protective circuit prevents the generated output voltage from rising above the maximum voltages. To achieve this, adjust the PGM and CNTRL pin of the IC BQ25505. In addition, the circuit is made to store energy in the secondary battery for use in an emergency. Three flexible input methods, including a single silicon photodiode, a solar cell, and an array of silicon photodiodes are used to harvest the energy from an ambient environment and provide input to the battery management unit. The battery management unit circuit layout with the array of silicon photodiodes (BPW34) is meant to detect light energy and generate an output voltage (which is the total of all the photodiode outputs) to power the device. A transimpedance amplifier circuit is used to convert light energy between 400 and 700 nm into voltage so that the battery management system can be powered. A single silicon photodiode and 5.5 V, 180 mA, 1 W solar cells are used in the battery management unit to detect light energy.

## 4. ECG Signal Processing and Feature Extraction

The signal to noise ratio has a major impact on signal quality. Noises of all kinds, including baseline drift, powerline interference, and high-frequency sounds, have a significant impact on the real-time ECG data capture [3,8]. These noises make it more difficult to accurately extract required information from an ECG signal. For processing and diagnosing purposes, it is therefore necessary to eliminate these noises [8,9]. The output of an instrumentation amplifier is fed into a filtering unit in this investigation. The developed module has a two-stage filtering unit. Low-pass and band-pass filters are used in the design of the two-stage filtering unit. The low-pass filter has a 100 Hz cutoff frequency built into its design. The band-pass filter is designed with the cut off frequency range of 0.5 to 100 Hz.

### 4.1. Wavelet-Based Signal Analysis

The desired time and frequency information which are necessary for extracting ECG features are obtained by applying wavelet transform to an ECG signal. Numerous researchers have thoroughly examined the application of wavelet transform to an ECG signal [8,9]. This paper proposes a wavelet packet analysis method for obtaining ECG features by removing repeated and overlapped data during each level of the decompositions.

#### 4.1.1. Discrete Wavelet Transform

**Figure 6:**
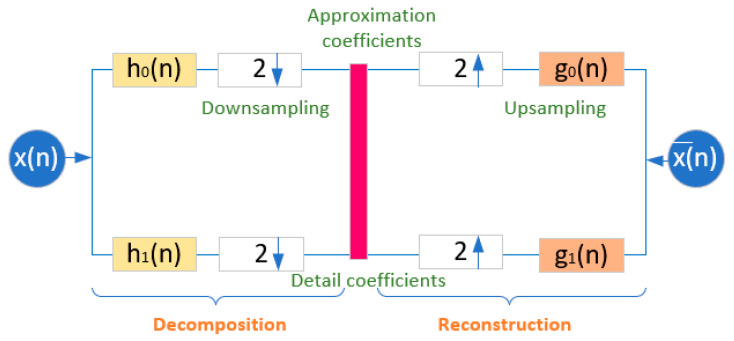
Wavelet decomposition and reconstruction principle.

Multiresolution analysis plays a crucial role in biomedical signals by offering varying resolution over a range of scales. The time or spatial range of the incoming bio signals is represented by the matching scale. The discrete wavelet transform is best utilized in the construction of multi-rate filter banks. As seen in Figure 6, it is composed of sequential low-pass and high-pass filters with a down-sampling factor of two. The wavelet transform provides further benefits over the short-time Fourier transform in avoiding the fixed-resolution issue. Additionally, it offers a superior frequency response over low frequency and a superior time resolution response over high frequency. In order to extract the required features for identifying numerous body vital factors, the wavelet transform is the ideal option for ECG signal processing.

Basically, the continuous wavelet transform (CWT) [3] of the input signal *x*(*t*) can be written mathematically as follows:

(1) $$X_{\omega }\left(a,\tau \right)=\frac{1}{\sqrt{a}}\int_{-\alpha }^{\alpha }x\left(t\right)\Psi^{*}(\frac{t-\tau }{a})dt$$

In Equation (1), the scaling parameter is represented by (*a*) and the translating parameter is represented by *τ*. The use of a scaling parameter in the equation is to dilate the signal with respect to its ranges. The low value of (*a*) provides contraction and the high value of (*a*) provides signal stretching. Also, the window location through the signal depends on the translating parameter *τ*. From Equation (1), it was observed that the scaling and translating of the mother wavelet *Ψ*(*t*), provides wavelet basis function, $\Psi \left(\frac{t-\tau }{a}\right).$ This can be mathematically expressed by the following:

(2) $$\Psi_{a,\tau }=\frac{1}{\sqrt{s}}\Psi (\frac{t-\tau }{a})$$

The working profile of the wavelet transform is described in terms of decomposing the original signal (*t*) into a basis function of time and scale ɸ(*t*) and the mother wavelet function *Ψ*(*t*).

(3) $$x\left(t\right)=\sum c_{j0k}{ɸ}_{jk}\left(t\right)+\sum \sum d_{jk}{ɸ}_{jk}\left(t\right)$$

where *j*_0_ represents the arbitrary starting scale. In Equation (3), $\sum c_{j0k}{ɸ}_{jk}\left(t\right)$ is called the approximation at scale *j*_0_ and the second parameter $\sum \sum d_{jk}{ɸ}_{jk}\left(t\right)$ is called the sum of the details. The mathematical representation of the approximation coefficients and the scaling function ${ɸ}_{j0k}\left(t\right)\;$is expressed by the following:

(4) $$c_{j0k}=\int x(t){{ɸ}^{*}}_{j0k}(t)dt$$

(5) $${ɸ}_{j0k}=\frac{1}{\sqrt{2^{j0}}}\;ɸ(\frac{t-k2^{j0}}{2^{j0}})$$

The mathematical expression for detail coefficients is given in Equation (6) and wavelet function is expressed in Equation (7).

(6) $$d_{jk}=\int x(t){{ɸ}^{*}}_{jk}(t)dt$$

(7) $${ɸ}_{jk}=\frac{1}{\sqrt{2^{j}}}\;ɸ\left(\frac{t-k2^{j}}{2^{j}}\right)$$

The optimum use of the discrete wavelet transform produced the decomposition of the original signal using low-pass and high-pass filters with the mother wavelet. The output of the decomposition is represented by approximation and detailed coefficients [8] which can be expressed by the following:

(8) $${A\cdot C}_{n,k}=\sum_{k=-\infty }^{k=\infty }x(k)g(2n-k))$$

(9) $${D\cdot C}_{n,k}=\sum_{k=-\infty }^{k=\infty }x\left(k\right)h(2n-k))$$

#### 4.1.2. Wavelet Packet Transform

**Figure 7:**
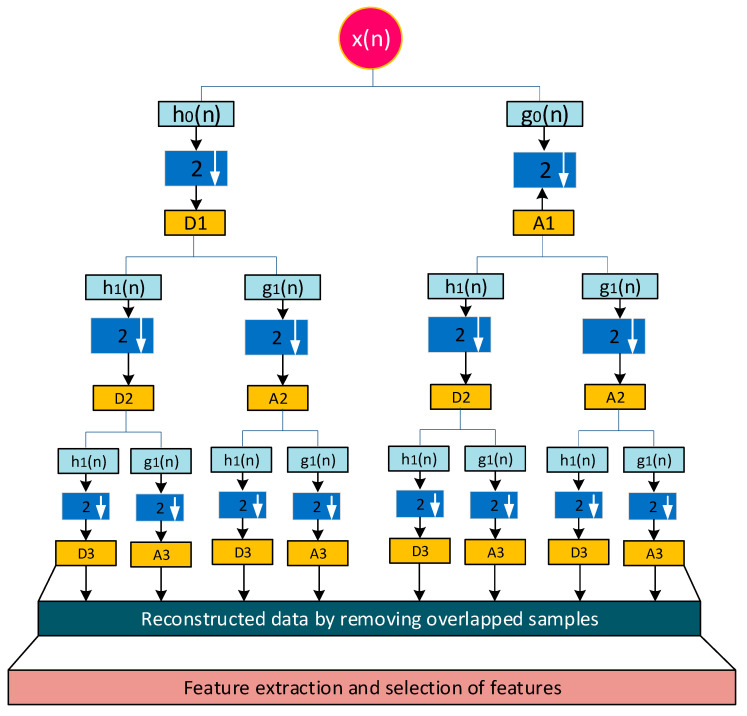
Discrete wavelet packet decomposition and reconstruction principle.

Each level of a discrete wavelet transform is computed by just using the low-pass and high-pass filters to pass the previous wavelet approximation coefficients. Here, the output of the wavelet packet transform is obtained by decomposing both the approximation and detail coefficients. The decomposition tree concept and procedures for decreasing repeated samples are depicted in Figure 7. Here, the signal is processed by low-pass and high-pass filters as mentioned by *h*_0_(*n*) and *g*_0_(*n*). Each stage of processed signals is further decomposed by the discrete wavelet packet transform and produces detail coefficients (D1) and approximation coefficients (A1). This work proposes and applies the Daubechies mother wavelet to the discrete wavelet packet transform on an ECG signal. In order to produce approximation and detail coefficients, the wavelet decomposition approach is employed up to four levels (D1–D4, A1–A4). The discrete wavelet packet transform-based algorithmic approach is more efficient due to synthesizing each low-pass and high-pass filter signal. The execution profile of this approach maintains lossless data during each level of decomposition. Therefore, this work mainly used the discrete wavelet packet transform to process the ECG signal.

#### 4.1.3. Selection of Mother Wavelets

Wavelet analysis presents an alternative enhanced windowing technique [3,9]. Long time periods are used in wavelet analysis to obtain low-frequency data. The wavelet families Morlet, Mexican hat, Haar, Daubechies, biorthogonal, coiflets, Symlets, and Meyer have all been demonstrated to be useful for analyzing ECG data [9]. The Daubechies wavelet families whose shapes approximate QRS complexes are the subject of this investigation. The lower frequencies are the focal points of the energy spectrum. Daubechies wavelets are used in this work to interpret ECG signals, identify peaks, and extract features.

#### 4.1.4. Machine Learning for ECG Classification

The identification and signal classification are a critical field of research in biomedical engineering. The classification and identification of numerous diseases through the function of statistical machine learning systems in the health sciences provides an effective way for the designer to develop an intelligent system [50,51,52]. Using a well-defined dataset as input and the proper training, disease categorization can be achieved. The process of selecting a machine learning method is primarily reliant on the input dataset’s accessibility and different signal characteristics. Both traditional machine learning and deep learning methods are commonly utilized for ECG signal classification [32,33,34,35,36,37,38,39,40,41,42,43,44]. This study suggests a number of machine learning approaches to be examined and determines which classifier is most suited to identify anomalies in the ECG. The machine learning algorithms are based on mathematical operations and linear statistical models. Numerous research works have been carried out over the years concerning the automated identification of aberrant ECG readings using hospital-based ECG data. The seven classifiers for ECG classification that are the focus of this work are the support vector machine, AdaBoost, random forest, gradient boosting, logistic regression, K-nearest neighbor, and bagging [53,54,55,56,57]. Also, the integration of random forest with deep decision tree architecture is tested with an input ECG signal.

## 5. Results and Discussion

### 5.1. ECG Signal Acquisition and Processing Through Analog Front-End System

**Figure 8:**
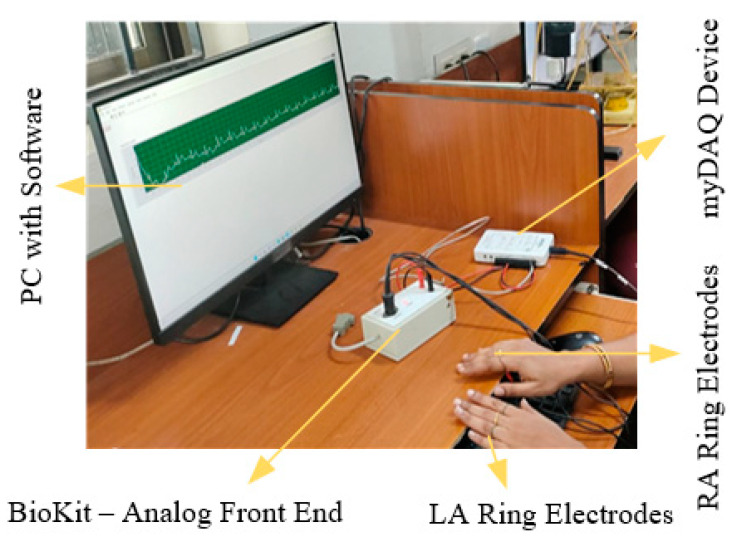
Experimental set up for acquisition of ECG signal.

ECG signals are acquired and analyzed through the development of systems built using an analog front-end system as shown in Figure 8. Three lead surface electrodes, an instrumentation amplifier, and filters are the basis of the developed device for acquiring ECG signals in real time. The three lead electrodes are attached with right arm, left arm and right leg and acquired the ECG signal.

**Figure 9:**
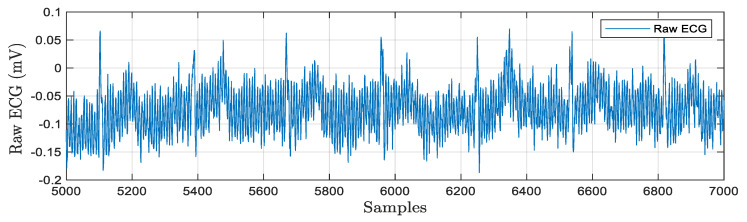
Acquired raw ECG signal through developed sensor.

**Figure 10:**
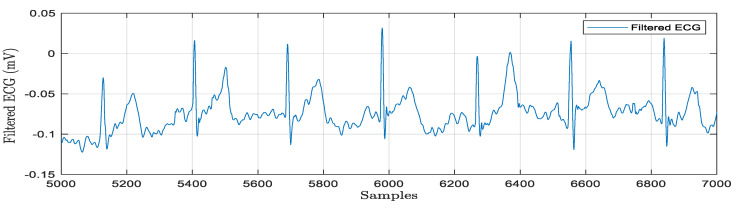
Processed ECG signal for baseline removal and feature extraction.

The real-time acquisition of a raw ECG signal is depicted in Figure 9. The electrodes are attached properly with electrode gel and the ECG signals are acquired for multiple patients. The sample rate is typically adjusted to match database signals. The acquired ECG signals can be stored as LVM or .xls files for offline analysis. Figure 3 shows the hardware design for a bio amplifier with a filtering unit. Here, baseline drift noise is considered, and digital filtering techniques have been designed in both hardware and software programming environments to process the signal for noise removal. The morphological and temporal properties of the ECG signal are impacted by the noise components. Noise reduction and feature extraction are accomplished through the use of filters and wavelet transform-based algorithms. LabVIEW 2014 and MATLAB R2018b programming languages are used to conduct the simulations. After the computing station receives a signal, the baseline wandering frequency components are eliminated using filtering techniques with a cut off frequency of 0.1 Hz–100 Hz. The obtained results are graphically represented in Figure 10.

**Figure 11:**
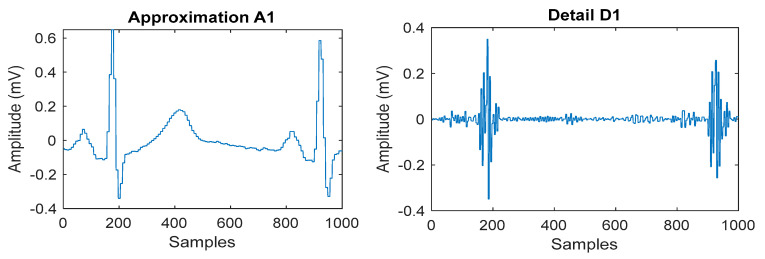
Level one wavelet decomposition of ECG signal.

**Figure 12:**
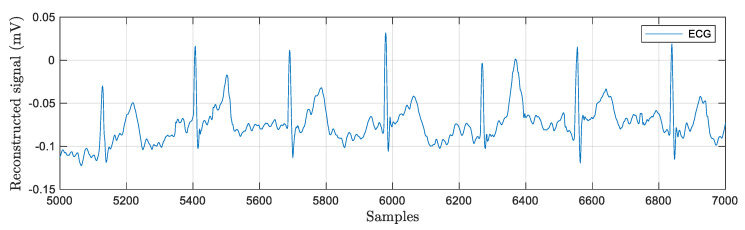
Reconstructed ECG signal through wavelet packet transform method.

**Figure 13:**
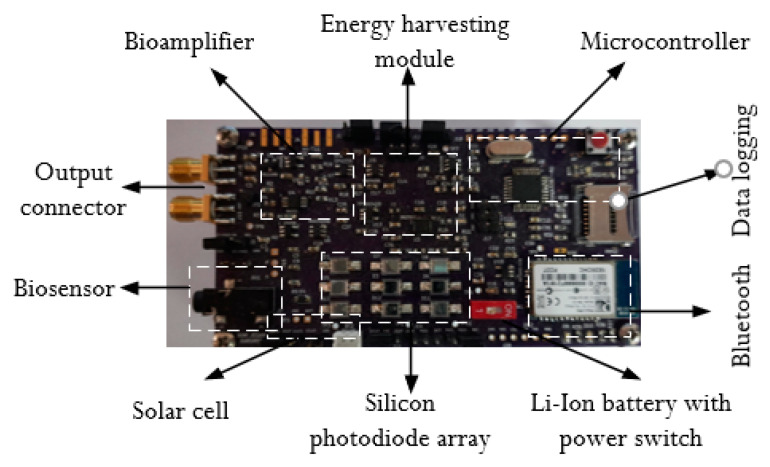
Developed self-powered ECG hardware.

**Figure 14:**
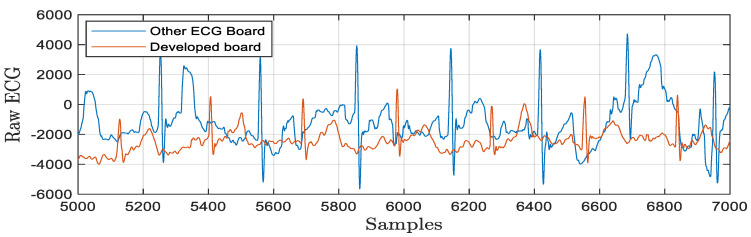
Performance of developed module with other ECG board.

The experimental technique for wavelet-based feature extraction from an ECG signal in real time has been the main focus of this work. Here, the ECG signal is processed by a low-pass and high-pass filter during each stage of wavelet decomposition using Daubechies wavelet. The output of each stage is further decomposed by the discrete wavelet packet transform and produced detail coefficients and approximation coefficients. The discrete wavelet packet transform-based algorithmic approach is more efficient due to synthesizing each low-pass and high-pass filter signal. The decomposed coefficients are produced at each stage of the wavelet packet decomposition up to four levels. The execution profile of this approach maintains lossless data during each level of decomposition by reducing overlapped samples at each stage. Ultimately, the reconstruction process aims to emulate an input signal while preserving all necessary information, producing output that is significantly superior to that of wavelet transforms. The Daubechies wavelet is employed in this instance with a four-level decomposition, allowing the information level to be increased in accordance with the signal’s needs. The results for the wavelet packet transform to obtain detailed and approximated coefficients are graphically represented in Figure 11. This algorithm has a good enough response to avoiding the unnecessary information that increases processing time. The signal is broken down into four levels in this instance, with the overlapped information at levels three and four being pushed to zeros. After that, it is rebuilt to create the signal with no information loss as shown in Figure 12. Low processing time and excellent temporal resolution are achieved by the developed method. The processed signal is further used for extracting the desired features which includes the mean and standard deviation of the heart rate and QRS complex and PR interval through an advanced statistical signal-processing tool kit. The performance of the developed board as shown in Figure 13 is compared with other available ECG analog front-end units by acquiring a raw ECG signal. The graphical representation of this comparison is depicted in Figure 14. The summary of the estimation of these features are represented in Table 6.

### 5.2. Battery Management Unit with Energy Harvesting

The developed energy-harvesting module provides flexible approaches for delivering power to the entire device. The energy is extracted from an ambient light by array of silicon photodiodes and solar cells and delivers the power to the device. The proposed energy-harvesting module with an array of silicon photodiodes and solar cells can operate under any sunlight irradiances to harvest solar energy for powering the device. The designed circuit is enabled to step up the output voltage to 4.2 V under indoor and outdoor light conditions with a minimum of 50 W/m^2^ irradiance. The proposed battery management unit with the energy-harvesting module is designed with a BQ25505 booster unit. Figure 5 shows the functional blocks involved for the construction of the battery management unit that can generate a stable output voltage of 4.2 V under various input voltages ranging from 0.2 V. Here, the light-sensing module is designed with eight numbers of tiny silicon photodiodes (BPW34) which are suitable for detecting the light intensity from 400 nm to 700 nm of visible wavelength from both indoor and outdoor light environments. The single photodiode produced a 1.1 V output through a current to voltage converter under 521 W/m^2^ of light intensity which was measured by solar meter. This voltage acts as an input to the battery management unit to generate an output voltage by harvesting the ambient light energy. The battery management unit can be operated by a minimum of 200 mV. In this study, an ECG analog front-end system is designed and integrated with an energy-harvesting module which is made up of low-power electronic components for collecting an ECG signal enabling long-life wearable applications. The overall size of the developed prototype is 10 cm (length) × 5.2 cm (width) which has provided full operation with a supply voltage of 3.3 V and ability to harvest the energy under both indoor and outdoor lighting conditions. The proposed system is designed with a newly designed energy-harvesting module that can collect solar power with very high efficiency to prolong the battery life under low-range indoor and outdoor light conditions. The developed circuit can be activated at a very low input voltage of ∼0.2 V. The proposed system can harvest energy even in indoor and outdoor light conditions and prolong the battery life under all lighting conditions. The experiments were carried out by acquiring the ECG signals and concluded that the developed device with a self-powered module could be the best solution for the home-based monitoring of an individual health status. Therefore, the proposed system can overcome the disadvantages of conventional wearable devices.

In addition, onboard power switching regulations are implemented in the developed device to deliver the power in a flexible approach for biosensing applications that provides novel circuit architectures and biosensing capability. A hardware prototype was constructed to show an energy efficient self-powered module as proof of a concept. It dramatically delivered the required power efficiently while keeping excellent accuracy for bio-signal monitoring. The experiments were carried out by acquiring the ECG signals and compared with commercially available devices as shown in Figure 14. The performance of the battery management unit as depicted in Figure 5 to generate the required output voltage under different light conditions is summarized in Table 7. The board is designed with low-power electronic components and suitable for wearable applications. The overall size of the developed prototype is 10 cm (length) × 5.2 cm (width) which has provided full operation with a supply voltage of 3.3 V and the ability to harvest energy under both indoor and outdoor lighting conditions.

### 5.3. ECG Signal Classification Using Machine Learning Techniques

**Figure 15:**
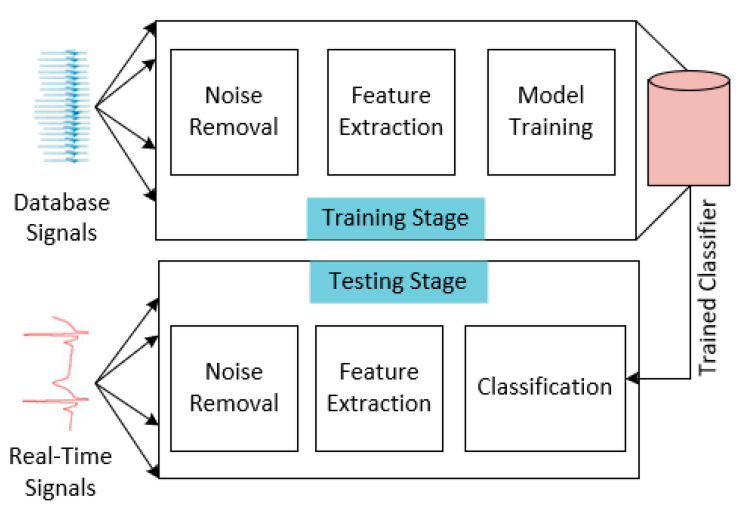
Functional blocks involved in proposed system for ECG classification.

The extracted features are used in our experimental investigation, and various machine learning techniques which are detailed in Section 3 are tested for abnormality detection and classification. Here, signals from the Sudden Cardiac Death Holter Database, INCART 2 Lead Arrhythmia Database, MIT-BIH Arrhythmia Database and MIT-BIH Supraventricular Arrhythmia Database [58] are also taken into consideration for training an ECG classification system. The set of databases and real-time signals are used to train the different classifier models. We conducted tests using the following classifiers: support vector machine, boosting, random forest, AdaBoost, logistic regression, K-closest neighbor, and bagging. The functional blocks of the developed system for ECG acquisition, processing, and classification are shown in Figure 15. The software algorithm has been developed and tested with databases and real-time signals. Random forest merges multiple decision trees to generate an efficient model that is more precise, efficient, and with low processing. It is frequently applied to tasks involving both regression and classification. By merging the predictions of separate decision trees, the random forest model is designed to enhance their performance and capacity for generalization. Using this approach, numerous decision trees are produced. From the dataset, this algorithm chooses n random samples. Random forest creates a unique decision tree for every set of samples. Each decision tree in a random forest divides the features into trees. Every decision tree determines the result. The accuracy will be high if there are many more decision trees. Algorithm 1 shows pseudo-code implementation for RF with decision tree classifier employed for ECG classification.
**Algorithm 1:** Pseudo Code for proposed random forest with decision trees [56,57]**Require:** Training sets: T(s) S—Input data X—FeaturesN—Decision trees **Random forest** (S,X) S_1 → S, selection of bootstrap sample N_I → (S_1_,X), Random tree learning **while** N = Randomly select subset of features (y) if N = Best feature splits from (y) then Random tree learning end if end while

The developed model is examined and contrasted with the performance of the chosen algorithms. Based on the findings as shown in Table 8, it was shown that the random forest algorithm combined with the deep decision tree technique performs better for ECG classification. The classification performance is improved by using a random forest classifier with more decision trees. The performance metrics used in this paper include accuracy, sensitivity, and specificity which can be estimated from true positive, false positive, false negative, and true negative [44]. The mathematical expression of these performance metrics is shown below.


(10) $$Accuracy\left(\%\right)=\frac{TP+TN}{TP+TN+FP+FN}\;\times \;100$$

(11) $$Sensitivity\left(\%\right)=\frac{TP}{TP+FN}\;\times \;100$$

(12) $$Specificity\left(\%\right)=\frac{TN}{TN+FP}\;\times \;100$$

The current study examined the effectiveness of dimensionality reduction strategies employing the wavelet packet transform approach on real-time and database signals. The discrete wavelet packet transform provides excellent performance, accurate measurement of decomposed coefficients, energy compaction, and reduction in sampling data. The current best strategy for creating a real-time system to improve classification accuracy has become more important. In this work, ECG signals are processed and classified using a hybrid random forest classifier in conjunction with multiple decision trees. The signals have been collected by developed devices and databases, and a machine learning algorithm is designed offline for classification. The latency from signal acquisition to classifications is about 5 s. The methods used in the experiments demonstrate how the characteristics of the ECG signal are used as inputs by several classifiers, and the system is trained using the same set of inputs. The 48 recordings in the MIT-BIH Database, the 75 records in the INCART 2 Lead Arrhythmia Database, and the 23 records in the Sudden Cardiac Death Holter Database comprise the ECG sources. Furthermore, according to the MIT-BIH Database, the gradient boosting classifier yields 97.76 percent accuracy, the AdaBoost classifier yields 96.86 percent accuracy, the logistic regression yields 94.85 percent accuracy, the KNN algorithm yields 98.78 percent accuracy, the SVM yields 96.30 percent accuracy, and the bagging classifier yields 98.77 percent accuracy.

The advantages of the proposed work for the development of a self-powered energy-harvesting electronic module and signal-processing framework for ECG signal classification are given as follows. The proposed methodology is utilized for the wavelet packet-based decomposition of ECG signals and feature extraction. The developed discrete wavelet packet transform-based approach for processing ECG signals successfully decreased the number of overlapping samples while cutting down on processing time. The ECG features from the signals of three lead ECGs have been recorded and tested with different machine learning algorithms. The working process of the proposed method does not require fine-tuning for achieving higher accuracy for ECG classification. The experiment result indicates that the proposed method can classify the ECG abnormality classes with 99.72 percent accuracy using the random forest with deep decision tree classifier. This work summarizes the comparison results of proposed work with other existing machine learning techniques. However, the results are significantly varying due to moderate change in dataset size, selection, and other signal-processing methods. The proposed model effectively classifies the abnormalities with 99.72% accuracy through defined training and testing data. The statistical performance indicators of the random forest with deep decision tree classifier and their comparison with other techniques are summarized in Table 8, containing the accuracy, sensitivity, and specificity. As a result, the proposed method used a different strategy to apply signal-processing techniques to extract the significant features from ECG data with minimal processing time by removing the overlapped samples. Also, the strategy used for classification requires minimal human effort, which gives additional strength to early prediction and analysis for many health-related problems. The comparison results show that the proposed method shows promise to become an effective way to diagnose arrhythmia disease for cardiologists by ECG signals. Numerous real-world applications, such as remote monitoring, patient monitoring, illness analysis and diagnosis, and cardiac devices, could be expanded upon by integrating through the proposed method.

## 6. Conclusions

This paper proposed the development of a self-powered portable electronic module with an integration of an onboard energy-harvesting facility for ECG signal processing and personalized health monitoring. The development of a multi-functional analog front-end system was presented which delivers a flexible approach to power the device through energy harvesting and a battery management unit and provides an easy acquisition of real-time bio signals. The experiments were carried out by acquiring the ECG signals and concluded that the developed device with the self-powered module could be a best solution for home-based monitoring of an individual health status. The dynamic multi-level wavelet packet decomposition framework has also been used in this paper to extract the desired ECG features. Further, a random forest with deep decision tree (RFDDT) architecture has been designed for ECG signal classification. The proposed signal-processing techniques effectively applied on the ECG signal for removing the noises. The dynamic discrete wavelet transform with 4 levels of decomposition stages is designed and applied on the ECG signal. The decomposed data form an ECG signal which contain the same repeated samples during each stage of decomposition further increase the data size and processing time. The proposed method contains a smart technique which allows to remove the repeated samples by zeroing the overlapped samples in their decomposition stages. The reconstructed signal maintains the original information with respect to input signals which are used for extracting ECG features. The proposed machine learning architecture based on random forest and decision trees are designed to classify ECG signals. Six classifiers are used to test the performance of the system. The performance of the proposed classification techniques was compared with an existing method and produced better performance for ECG classification. From the results, the random forest with decision trees architecture achieved the maximum accuracy of 99.72%. Future studies should include a different dataset in order to create multi-class classifiers for ECG classification.

## Figures and Tables

**Table 1:** Major ECG noises and frequency range.

ECG Noises	Sources	Frequency
Motion artifact	Electrode skin interface	1–10 Hz
Baseline wandering	Respiration	<1 Hz
Powerline interference	Load coupling	50/60 Hz

**Table 2:** Electrical activity of heart and its wave features.

Electrical Activity	Features
Atrial activity	P wave F wave QT interval P wave amplitude F wave interval
Ventricular activity	QRS complex onset and offset peaks RR interval R peak Peak arrival time

**Table 3:** Summary of features extracted from RR interval.

Domain Type	RR Interval Features
Time	Average of RR intervals, root mean square, histogram, median, average deviation, maximum deviation, number of successive RR pairs, change in RR intervals, interpolation of RR intervals histogram, HR mean, QRS amplitude mean
Frequency	ULF, VLF, low frequency power, high frequency power, frequency ratio, power spectral density
Statistical	Mean, median, standard deviation, kurtosis, range, skewness

**Table 4:** Summary on ECG classification techniques.

Ref.	Classification Techniques	Accuracy
[32]	Genetic algorithm–support vector machines	96.00%
[33]	PSO-SVM	91.75%
[34]	PCA, LDA, ICA and neural network	99.28%
[35]	Back propagation, genetic algorithm	98.77%
[36]	Support vector machine (SVM)	98.46%
[37]	AdaBoost ensemble classifier	99.28%
[38]	One-against-one (OAO-SVM)	94.50%
[39]	Linear SVM, random forest	85.58%
[40]	Echo state networks	92.70%
[41]	LSTM, convolutional neural network	87.50%
[42]	Sigmoid and SVM classifier, CNN and LSTM	94.27%
[43]	Bayesian belief network	94.27%
[44]	Bidirectional LSTM, random forest classifier	98.84%

**Table 5:** Sources and summary of ECG wave patterns.

ECG Segment	Sources	Duration (ms)
P wave	Atrial depolarization	60–80
QRS complex	Ventricular depolarization	80–120
PR interval	Atrial depolarization plus AV nodal delay	120–200
ST segment	Isoelectric period of depolarized ventricles	100–120
T wave	Ventricular repolarization	120–160
QT interval	Length of depolarization plus repolarization	>0.44
RR interval	Distance between R Peaks	0.6–1.2 s

**Table 6:** ECG signal extracted features.

Features	Tachycardia	Bradycardia	Normal
HR mean	120	84.09	78.06
QRS amplitude mean	1.184	0.636	0.86
QRS time mean	0.143	0.058	0.06
PR interval mean	0.126	0.141	0.15
HR SD	0.98	0.85	0.86
QRS amplitude SD	0.011	0.018	0.02
QRS time SD	0.008	0.002	0.002
PR interval SD	0.023	0.012	0.009

**Table 7:** Evaluation of battery management unit to generate output voltage under different light intensity.

S. No.	Light Intensity (W/m^2^)	Silicon Photodiode/Solar Cell Amplifier Output (V)	Battery Management Unit				
			R1	R2	R3	R4	R5
			5.62 MΩ	7.32 MΩ	5.62 MΩ	5.49 MΩ	1.9 MΩ
1	1134	3.3	Output voltage generated = 4.2 V				
2	956	3.1					
3	834	2.7					
4	752	2.4					
5	521	1.1					
6	348	0.9					

**Table 8:** Performance comparison of machine learning techniques [53,54,55,56,57,58].

Methods	MIT-BIH Arrhythmia Database			MIT-BIH Supraventricular Arrhythmia Database			INCART Lead 2 Arrhythmia Database			Sudden Cardiac Death Holter Database		
	Acc (%)	Sen (%)	Spec (%)	Acc (%)	Sen (%)	Spec (%)	Acc (%)	Sen (%)	Spec (%)	Acc (%)	Sen (%)	Spec (%)
Gradient	97.76	94.92	98.04	96.21	88.20	97.16	99.35	98.76	99.44	97.87	87.18	98.63
AdaBoost	96.86	88.95	97.66	95.51	87.17	96.44	99.24	98.26	99.38	97.52	86.48	98.27
Logistic	94.85	86.78	95.45	93.08	80.42	94.22	98.64	97.48	98.80	96.31	88.51	96.67
KNN	98.78	97.45	98.92	97.53	92.41	98.17	99.62	99.19	99.68	98.01	87.34	98.79
SVM	96.30	94.59	96.43	95.53	88.66	96.27	99.23	98.01	99.40	97.39	88.09	97.97
Bagging	98.77	96.21	99.05	97.85	92.14	98.56	99.63	98.81	99.75	98.12	87.49	98.91
RFDDT	99.01	97.66	99.15	98.03	93.44	98.63	99.72	99.17	99.79	98.28	88.80	98.98

## Data Availability

The original data presented in the study are openly available in MIT-BIH Arrhythmia Database at https://physionet.org/content/mitdb/1.0.0/ (accessed on 13 April 2024) and MIT-BIH Supraventricular Arrhythmia database at https://physionet.org/content/svdb/1.0.0/ (accessed on 13 April 2024) and Sudden Cardiac Death Holter Database at https://physionet.org/content/sddb/1.0.0/ (accessed on 13 April 2024) and St Petersburg INCART 12-lead Arrhythmia Database at https://physionet.org/content/incartdb/1.0.0/ (accessed on 13 April 2024).
